# Field-Level Sublethal Effects of Approved Bee Hive Chemicals on Honey Bees (*Apis mellifera* L)

**DOI:** 10.1371/journal.pone.0076536

**Published:** 2013-10-18

**Authors:** Jennifer A. Berry, W. Michael Hood, Stéphane Pietravalle, Keith S. Delaplane

**Affiliations:** 1 Department of Entomology, University of Georgia, Athens, Georgia, United States of America; 2 School of Agricultural, Forest, and Environmental Sciences, Clemson University, Clemson, South Carolina, United States of America; 3 The Food and Environment Research Agency, Sand Hutton, North Yorkshire, United Kingdom; Ghent University, Belgium

## Abstract

In a study replicated across two states and two years, we tested the sublethal effects on honey bees of the miticides Apistan (tau fluvalinate) and Check Mite+ (coumaphos) and the wood preservative copper naphthenate applied at label rates in field conditions. A continuous covariate, a colony Varroa mite index, helped us disambiguate the effects of the chemicals on bees while adjusting for a presumed benefit of controlling mites. Mite levels in colonies treated with Apistan or Check Mite+ were not different from levels in non-treated controls. Experimental chemicals significantly decreased 3-day brood survivorship and increased construction of queen supercedure cells compared to non-treated controls. Bees exposed to Check Mite+ as immatures had higher legacy mortality as adults relative to non-treated controls, whereas bees exposed to Apistan had improved legacy mortality relative to non-treated controls. Relative to non-treated controls, Check Mite+ increased adult emergence weight. Although there was a treatment effect on a test of associative learning, it was not possible to statistically separate the treatment means, but bees treated with Apistan performed comparatively well. And finally, there were no detected effects of bee hive chemical on colony bee population, amount of brood, amount of honey, foraging rate, time required for marked released bees to return to their nest, percentage of released bees that return to the nest, and colony Nosema spore loads. To our knowledge, this is the first study to examine sublethal effects of bee hive chemicals applied at label rates under field conditions while disambiguating the results from mite control benefits realized from the chemicals. Given the poor performance of the miticides at reducing mites and their inconsistent effects on the host, these results defend the use of bee health management practices that minimize use of exotic hive chemicals.

## Introduction

The parasitic honey bee mite, *Varroa destructor* Anderson and Trueman has been responsible for transitioning beekeeping from one of the world’s most chemical-averse agricultural industries to one of its most chemical-dependent. In the United States, the synthetic acaricides tau-fluvalinate (Apistan™) and coumaphos (Check Mite+™) are routinely used to control this exotic honey bee pest. It is generally believed that Varroa-related losses would be unacceptably high without these inputs. Although these products have low acute toxicity (high LD_50_s) to honey bees, there is growing evidence that they are not entirely benign. Rinderer et al. [1] showed that drones exposed to fluvalinate during immature development have increased mortality and reduced body weight and tend toward lower sperm counts, and Burley et al. [2] showed that drones similarly exposed to coumaphos have lower sperm viability. Haarmann et al. [3] showed that queens have reduced body weight if reared in the presence of elevated levels of fluvalinate. At beeswax coumaphos levels equal to the legal tolerance of 100 ppm >50% of queen cells were rejected by nurse bees in a rearing colony, and those queens that survived to adulthood weighed less than control queens [4] and at 6 months expressed only 31% survival compared to control group survival of 48% [5]. Coumaphos has been shown to alter honey bee gene expression for detoxification pathways and may down-regulate gene products associated with cellular or humoral immunity [6]. There is evidence that acaricides alter physiological functions, immune responses, and detoxification functions in the host bees rendering them more susceptible to pathogens and pesticides [7–6]. And finally, the active ingredients fluvalinate and coumaphos have been shown to synergize in the company of each other, elevating the honey bee toxicity of each to potentially injurious levels [8]–[9].

Honey bee exposure to toxins has been a subject of increasing scrutiny as colony numbers continue to decline in the United States and Europe [10]–[11]. Survey analyses of bees and hive matrices show a high degree of pesticide exposure, both in diversity of compounds and level of residues [12]–[13]. But it has not proven easy to assign direct causation to pesticides or to any single factor, and the prevailing thinking is that bee decline is a product of many interacting stressors including but not exclusive to environmental toxins [14]–[15]. Field pesticide symptoms sometimes lack clear indication, raising interest in sublethal effects on bees – morbidities that escape casual observation but nevertheless add up to colony-killing effects. The fact that beekeeper-applied chemicals top the list of compounds found in hive matrices [12], [13], [16] underscores the need to examine these chemicals for their sublethal effects and potential contributions to bee health problems.

In this paper we report a two-year (2008, 2009) study replicated across two states (Georgia, South Carolina) looking for sublethal effects on bees at labeled rates of compounds registered for use by beekeepers in the United States: the synthetic acaricides tau-fluvalinate (Apistan™) and coumaphos (Check Mite+™) used to control Varroa mites and, in Georgia only, copper naphthenate (Jasco™) used to protect wooden hive parts from termites and decay fungi. Key to our purposes was a simultaneous statistical control for the effects (presumably beneficial) of the miticides Apistan and Check Mite+. In other words, we wanted to parse out the benefits of miticides so that we could unambiguously examine them for their sublethal effects on the insects they are designed to protect. We did this by tracking colony mite level with three independent measures and using a combined colony mite index score as a covariate with the fixed effect colony chemical treatment. This implies the non-controversial assumption that mite depredations are lower in colonies in which mites are controlled with miticides.

## Materials and Methods

### General Set-up and Field Measurements

Experimental bee colonies were placed on lands owned and maintained by the University of Georgia or Clemson University for the explicit purpose of research, so no special permissions were required. No endangered or threatened species were involved in these studies.

Forty eight experimental colonies were set up in Georgia (4 treatments×12 replicates) and 24 in South Carolina (4×6), each colony consisting of a single 10-frame Langstroth hive body, a queen excluder, and enough honey supers to accommodate incoming nectar. We took pains to eliminate incipient pesticide residues as sources of variation. All hives were begun with factory-new equipment, and instead of using beeswax comb foundation (a potential source of exotic residues) we fitted each frame with a 2.5-cm strip of wax-less plastic comb foundation across the top bar. Bees used this plastic strip as a template to construct semi-natural combs inside the wooden frames. Within state, each colony was randomly selected to receive one of the following treatments at labeled rates: (1) two strips of Apistan inserted one between frames 3 and 4 and one between frames 5 and 6 for 42 days, (2) two strips of Check Mite+ inserted one between frames 3 and 4 and one between frames 5 and 6 for 42 days or (3) no treatment, and in Georgia only (4) a 37.5×45.7×0.3-cm sheet of wood laminate board impregnated with 2% copper naphthenate solution and placed on the hive floor for 42 days to simulate bee exposure to treated woodenware. After year 1 we sampled experimental colonies for target chemical residues in beeswax. We took wax from brood frames in position 1 or 10, representing the furthest possible distance from the experimental chemicals. The samples were sent to the USDA AMS National Science Laboratory in Gastonia, NC for chemical residues analysis for the target active ingredients shown in Table 1. In year 2, colonies retained their original treatment designations, and those that died were re-started with combs of the appropriate treatment saved in a freezer from the previous year.

**Table 1:** Beeswax chemical residue analysis (ppb) in experimental colonies after first season.

Georgia					
	Experimental treatments				
Active ingredients screened	Non-treated	Apistan™	Check Mite+™^c^		Cu naphthenate
			Year 1	Year 2	
2,4-dimethylaniline^a^	ND	ND	ND	ND^d^	ND^d^
2,4-dimethylphenyl formamide^a^	ND	ND	ND	ND	ND
Amitraz^a^	ND	ND	ND	ND	ND
Coumaphos^b^	392	429	256,000	514,000	212
Fluvalinate^b^	18	16,600	219	1700	trace
Elemental Cu	NA	NA	NA	NA	58.5
**South Carolina**					
**Active ingredients screened**	**Non-treated**	**Apistan™**	**Check Mite+™** c		**Cu naphthenate**
2,4-dimethylaniline^a^	ND	ND	ND		NA
2,4-dimethylphenyl formamide^a^	ND	ND	ND		NA
Amitraz^a^	ND	ND	ND		NA
Coumaphos^b^	9310	24.7	271,000		NA
Fluvalinate^b^	ND	3290	ND		NA

a detection limit (ppb) 4.0.

b detection limit (ppb) 1.0.

c Check Mite+™ colonies in Georgia were sampled both years to confirm unexpecedly high residues in year 1.

d detection limit (ppb) 50.

Each year we measured relative colony Varroa mite levels using three methods: (1) mites per 100 bees recovered from strained alcohol samples, (2) natural 24-hr mite drop recovered from hive floor inserts, and (3) mites recovered from hive floor inserts during 24-hr after dusting colony with 250 mL powdered sugar sifted onto top bars of brood frames. Measurements with one or more of these methods were done in Jul, Aug, Sep, Oct, and Nov 2008 and Apr, Jun, Aug, Sep, Oct, Nov, and Dec 2009.

In each state in each year, chemical applications were applied and data collected in each of two seasons: spring (May–Jun) and late-season (Aug–Sep). The following dependent field measurements were taken after a 42-day treatment interval: (1) brood survivorship, (2) number of queen cells in construction, (3) frames of adult bees, (4) frames of brood, (5) frames of honey, (6) foraging rate, (7) time for marked, released bees to return to the nest, (8) percentage of marked, released bees that return to the nest, and (9) incidence of “medium”, “medium high”, or “high” colony levels of *Nosema* spp. spores.

Frames of brood, bees, or honey were derived by taking the mean of results from two independent observers who visually estimate the surface area of comb surfaces covered by each target [17]–[18]. Brood survivorship was measured by placing a sheet of transparent acetate onto a brood comb and marking on the acetate the location of 40 cells of live, uncapped brood. Combs were selected that were not in direct contact with pesticide strips. Three days later, the same sheet of acetate was returned to the same frame and surviving brood counted to determine percentage brood survivorship. Bee foraging rates at the colony entrance (number of bees exiting per min) was measured during days of good flight condition. The number of queen cells under construction was noted regularly when colonies were opened and summed for each colony. Time (sec) for released bees to return to the nest and percentage of released bees that return to the nest were derived with a mark-recapture technique. Twenty-five foraging-age bees from each colony were collected at the hive entrance, narcotized in the field with either CO_2_ or ice, and marked on the thorax with a colony-specific color. One person moved the marked bees to a site 0.5-km away, and using cell phones communicated release time to observers back at the hives who then observed hive entrances for 15 min and recorded the number and time for each returning marked bee. Colony Nosema levels were determined by sampling 25 adult bees in alcohol and microscopically examining each for Nosema spores in macerated and suspended abdmoninal tissue [19]. Because Nosema spore distribution tends to be heavily clumped – large numbers in a few individuals [20] – we subjectively assigned each bee into one of five classes: (1) no spores, (2) low number of spores, (3) medium, (4) medium high, or (5) high. We analyzed and report our results as the incidence of colony Nosema levels scoring “medium,” “medium high,” or “high.”

### Conditioned Learning and Memory

Conditioned learning response and memory retention were tested with the Proboscis Extension Reflex (PER) assay [21]. A test arena was constructed to direct air over a scent and onto the face and antennae of a tethered bee which the operator could observe and reward with a drop of sugar syrup onto antennae or mouth parts. An aquarium pump was used to direct scented air onto the bee and an exhaust fan to vent it away. Individuals were restrained in a plastic drinking straw with only the mouthparts and antennae free. Bees reflexively extend the proboscis when their antennae are touched with a droplet of sucrose solution. If this process is accompanied with an odor stimulus, it sets the stage for a test of associative learning. There were three phases to the assay: the conditioning (learning) phase, a test blank, and the testing (memory) phase [22]. For each bee there were five conditioning trials during which the bee was restrained, a conditioning stimulus of odor (geraniol) blown across its face for 6 sec, a droplet of sucrose applied to the antennae after 3 sec, and the bee thereafter rewarded with the same droplet to the proboscis. A bee responding (extending proboscis) to the stimulus at or after the second trial was considered to be expressing a learned conditioned response; individuals were eliminated who extended the proboscis in trial 1 before the reward because there was no reason to expect conditioned learning at this point. The five conditioning trials were followed by a blank test of plain air to eliminate individuals responding to the mechanical stimulus of forced air. Individuals that exhibited conditioned learning in the first experiment were retained for four post-conditioning test intervals to appraise memory: 7-min post conditioning, 14-min [23], 28-min, or 56-min. In both conditioning and trial phases the response variable was percentage of individuals extending the proboscis. Thus, the assay measures expression of both learning and memory.

### Adult Bee Emergence Weight and Longevity

Emergence weight and daily cumulative mortality were compared for adult bees reared as immatures in field colonies under the chemical regimes. One frame of teneral adults emerging from their cells was removed from each colony and bagged overnight to collect newly-emerged bees. The next day, newly emerged bees from each colony were weighed and placed into colony-specific cages (target 50 bees per cage, *n* = 24 cages) and housed in an incubator at 35°C. Bees were fed sugar syrup, water, and pollen *ad libitum*. Cumulative daily mortality was counted until the last bee died.

### Statistical Analyses

All analyses were performed with the GenStat 15.1 statistics package [24]. In cases such as emergence weight, average numbers of frames of bees, brood, honey or the time taken to return to the apiary, the data were analyzed by analysis of variance recognizing colony mite level as a covariate and chemical hive treatment as fixed effect. The mite covariate was created by combining with a *Z* transformation into one synthetic mite index the three relative measures of colony mite level: (1) mites per 100 bees recovered from alcohol samples, (2) natural 24-hr mite drop, and (3) mites recovered after dusting with powdered sugar. When response data were counts (proportions), they were analyzed using a Generalized Linear Model (GLM) [25] assuming a Poisson (binomial) distribution and using a logarithm (response logit: log(p/(1−p))) link function. When a significant (*P*≤0.05) effect of treatment was detected, the treatments were compared using means separating groupings. The predictions and 95% confidence intervals were produced for each treatment for an average mite level based on the range of mite levels observed in the population.

The effect of treatment on colony mite measures was tested with a simple analysis of variance recognizing treatment as fixed effect after log-transforming the colony mite measures to correct for the skewness of the data.

For the PER learning analysis, the data were first sorted to the level of year, state, season in which test was performed (summer or fall), chemical treatment, and learning trial (time effect) in order to create independent replicates. The conditioning trials were repeated on the same individuals over five trials; therefore it was necessary to account for repeated measures. Because the data also had a binomial structure (X respondents out of Y tested), we used Generalized Estimated Equations (assuming a binomial distribution) [26], with a logit link function. The effects of treatment and trial (and their interaction) were tested using a *Χ^2^* test based on the change of deviance between models.

For the PER memory analysis, the data were number of respondents out of the number of individuals tested. Therefore, it was still necessary to account for the binomial distribution of the data. However, there was only one observation per bee, so it was not necessary to account for repeated measures. As a result, a Generalized Linear Model with a logit link function was used. The effects of treatment, post-conditioning test interval and their interaction were tested through *F* tests looking at deviance ratios.

When looking at cumulative adult mortality, the data were analyzed using a proportional hazard model for comparing the overall (i.e. whole pattern) mortality of the bees in the different treatment groups. The Cox proportional hazard model [27] relies on the notion of hazard function (in other words, estimated risk of death), defined as the probability of an individual dying at a fixed time point given that the individual has survived up to that point. This hazard function is defined piece-wise and uses each time point when death is recorded for any of the treatments and assumes that the baseline hazard is constant between two consecutive time points. One assumption made by the Cox proportional hazard model is that the bees in the “Control” group have a baseline hazard function and that the bees in the other treated groups have a hazard function proportional to it. Although the original ratios from the Cox proportional hazards model represent the increase (or decrease) in mortality of the insects in the different treatment groups in comparison to the control group, it is possible to compare those ratios to one another and therefore compare individual hazards to one another.

## Results and Discussion

In this study we attempted to identify sublethal effects on bees from field label rates of in-hive chemicals commonly used by beekeepers in the United States. The challenge was to do this while controlling for health benefits presumably derived from using these chemicals to control mites. We attempted to control this confounding variable by constructing a continuous covariate – a mite index score – from three independent measures of relative colony mite level. This covariate strengthens our argument that the colony strength measures reported below are relatively unambiguous indicators of the effects of these chemicals on the insects they are designed to protect.

Chemical residue analysis of brood comb wax after the first season confirms that the experimental active ingredients were the predominate exposures in their respective test colonies (Table 1). However, it was not unusual for low levels of non-target active ingredient to occur. For example, whereas fluvalinate was predictably the predominate exposure in Georgia colonies receiving Apistan™ (16,600 ppb), there were also detectable amounts of coumaphos (429 ppb). As no beeswax foundation was used to start these colonies, these exotic residues are likely the work of drifting bees or other unknown environmental exposures. More surprising was the high levels of coumaphos detected in colonies treated with Check Mite+™ at label rates. Comb residues of coumaphos at the end of year one in both Georgia and South Carolina were over five times the EPA tolerance of 45,000 ppb (Table 1). We analyzed Georgia Check Mite+™ colonies again at the end of year two, and residues had more than doubled after a second season’s use. These high residues are unexpected given that (1) treatments were applied at label rates, (2) experimental chemicals were not in hives at time of sampling, and (3) samples were taken from brood combs at the edge of the brood super and furthest from the site of treatment. Analytic standards were not available for copper naphthenate, but elemental copper was predictably detected in colonies receiving the wood preservative in Georgia.

### Field Measurements: Colony Varroa Levels

Results are shown in Table 2. Chemical hive treatment had significant effects on natural 24-hr colony mite drop and powder sugar-assisted mite drop (Table 2), but no significant effects on mites per 100 bees (*P* = 0.36). The miticidal properties of Apistan and Check Mite+ were weak or not evident, in neither case differing from non-treated controls; this is consistent with evidence for Varroa resistance to both these chemicals in the United States [28]–[29]. It is worth noting, however, that mite control although never different from non-treated controls was numerically optimized in colonies receiving Apistan™ (Table 2). With both measurements mite levels were significantly lower in colonies treated with the miticide Apistan than in colonies treated with the wood preservative copper naphthenate.

**Table 2:** Effects of field-rate in-hive chemical treatments on colony Varroa mite measures.

analysis^a^	treatment	mean^b^	*n*	min	Q1	median	Q3	max
Mite drop natural^c^								
*F*_tmt_ = 3.3; df = 3,186; *P* = 0.023	Non-treated	8.1 ab	55	0	1.0	7.5	41.6	200.9
	Apistan™	4.8 a	53	0	0.1	4.1	24.2	145.7
	Check Mite+™	7.5 ab	52	0	1.2	8.3	31.1	143.9
	Cu naphthenate	15.9 b	30	0.8	6.3	15.4	45.8	89.2
Mite drop sugar^d^								
*F*_tmt_ = 2.8; df = 3,158; *P* = 0.040	Non-treated	15.1 ab	47	0	1.6	14.0	66.4	523.8
	Apistan™	12.1 a	46	0	2.0	15.0	60.0	336.8
	Check Mite+™	13.2 ab	42	0	3.0	12.5	66.0	411.3
	Cu naphthenate	40.3 b	27	3.6	14.6	44.6	108.5	347.7

a *F*_treatment_ = *F*_tmt_.

b Means separated by Tukey’s 95% confidence intervals on the log-transformed data (log(value+1)). However, for convenience we here show mean separations on the back-transformed predicted means.

c Mites recovered per 24 hr from hive floor inserts.

d Mites recovered from hive floor inserts after dusting colony with powdered sugar.

It is important to note here that when the mite covariate was significant in the results reported below, the direction of the effect was always negative such that increasing mite levels were associated with decreasing measures of colony strength, with one exception – time for marked bees to return to the nest. Mite levels were therefore important in these measures, but it seems that mite levels varied independently of the experimental chemicals, two of which were commercial miticides.

### Field Measurements: Colony Strength Measures Adjusted for Mite Level

In all these dependent variables the effect of colony mite level was controlled as an independent covariate, and when the effect was significant it is shown in Table 3. There were no significant effects of chemical hive treatment nor mite covariate on frames of honey, foraging rate, and percentage of marked released bees that return to the nest (*P*>0.05).

**Table 3:** Effects of field-rate in-hive chemical treatments on honey bee biometrics.

analysis^a^	treatment	*n*	lower CI	predicted mean^b^	upper CI	
Brood survivorship^c^						
*F*_tmt_ = 4.5; df = 3,178; *P* = 0.004	Non-treated	54	94.1	96.0 b	97.3	
*F*_mite_ = 2.5; df = 1,178; *P* = 0.12	Apistan™	49	90.2	92.7 a	94.6	
	Check Mite+™	51	90.6	93.0 a	94.8	
	Cu naphthenate	29	86.3	90.1 a	92.9	
Queen cells started						
*F*_tmt_ = 5.6; df = 3,121; *P* = 0.001	Non-treated	34	0.15	0.47 a	1.51	
*F*_mite_ = 0.6; df = 1,121; *P* = 0.43	Apistan™	32	1.88	3.02 b	4.85	
	Check Mite+™	29	2.16	3.44 b	5.49	
	Cu naphthenate	31	1.31	2.28 b	3.97	
Frames of bees						
*F*_tmt_ = 1.1; df = 3,128; *P* = 0.33	Non-treated	40	4.77	5.71	6.65	
*F*_mite_ = 36.3; df = 1,128; *P*<0.001	Apistan™	37	3.74	4.72	5.70	
*F*_tmt*mite_ = 3.1; df = 3,128; *P* = 0.030	Check Mite+™	36	4.78	5.76	6.74	
	Cu naphthenate	23	4.52	5.75	6.98	
Frames of brood						
*F*_tmt_ = 0.9; df = 3,128; *P* = 0.45	Non-treated	40	1.92	2.32	2.73	
*F*_mite_ = 42.9; df = 1,128; *P*<0.001	Apistan™	37	1.51	1.94	2.36	
*F*_tmt*mite_ = 4.2; df = 3,128; *P* = 0.008	Check Mite+™	36	1.97	2.40	2.82	
	Cu naphthenate	23	1.55	2.09	2.62	
Time (sec) to return to nest^d^						
*F*_tmt_ = 2.5; df = 3,50; *P* = 0.07	Non-treated	20	427.6	481.5	535.5	
*F*_mite_ = 8.5; df = 1,50; *P* = 0.005	Apistan™	16	364.9	426.1	487.2	
Covariate estimate: −176.1±59.4	Check Mite+™	12	478.0	547.7	617.4	
	Cu naphthenate	7	352.4	446.7	541.0	
Incidence of Nosema spore load scoring “medium,” “medium high,” or “high”^e^						
*F*_tmt_ = 2.0; df = 3,51; *P* = 0.13	Non-treated	15	0.5	1.4	3.8	
*F*_mite_ = 5.2; df = 1,51; *P* = 0.027	Apistan™	14	0.4	1.2	3.5	
Covariate estimate: 1.9±0.7	Check Mite+™	13	0.5	1.5	4.1	
	Cu naphthenate	14	2.2	4.1	7.6	
Percentage bees learning^f^						
*X^2^*_tmt_ = 20.6; df = 3; *P*<0.001	Non-treated	28	9.9	12.9	16.8	
*X^2^*_time_ = 15.5; df = 3; *P* = 0.0015	Apistan™	24	11.1	14.2	17.9	
*X^2^*_tmt*time_ = 8.1; df = 9; *P* = 0.52	Check Mite+™	28	9.9	12.7	16.2	
	Cu naphthenate	8	9.4	12.3	15.9	
Adult emergence weight (mg)						
*F*_tmt_ = 3.5; df = 3,67; *P* = 0.020	Non-treated	22	97.5	100.6 a	103.7	
*F*_mite_ = 10.5; df = 1,67; *P* = 0.002	Apistan™	16	100.4	104.0 ab	107.7	
Covariate estimate: −9.6±3.2	Check Mite+™	20	103.7	106.9 b	110.1	
	Cu naphthenate	14	103.3	107.2 b	111.1	

a Accumulated analysis of deviance, *F*_treatment_ = *F*_tmt_. Three independent measures of colony mite level were taken (the two shown in Table 2+ mites per 100 bees recovered from strained alcohol samples) and combined by *Z* transformation into one covariate term *F*_mite_.

b When presented, mean separation groups are derived on the transformed scale, but for convenience we here show means on the back-transformed scale.

c Percentage of open brood cells surviving 3 d.

d Time (sec) for bees to return to nest from release site 0.5 km from nest within 30 min.

e Proportion of bees from a colony sample of *n* = 25 falling into subjective classes of “medium,” “medium high,” or “high” numbers of *Nosema* spp. spores.

f Percentage bees exhibiting conditioned learning response in Proboscis Extension Response assay. Each bee within chemical treatment was tested in 4 successive conditioning (learning) trials with the expectation that this would discriminate earlier versus delayed learning; data for trial 1 were discarded because there was no reason to expect individuals responding at the first trial to be exhibiting a conditioned response.

Brood survivorship was significantly affected by hive chemical regime, whilst it was not significantly affected by the mite covariate. Brood survivorship was significantly higher in non-treated controls than in colonies receiving bee hive chemicals. These results provide context to the work of Wu et al. [30] who housed bees on brood combs with a known history of high pesticide residues or on combs that were relatively non-contaminated. The “high” combs contained an average of ten different pesticide residues, the three most common being fluvalinate, coumaphos, and coumaphos oxon – a breakdown metabolite. Although brood survivorship was not different between the two comb types, these authors detected delayed larval development in young bees reared on the “high” residue combs. Our present data suggest that negative effects such as these translate into reduced larval survivorship with bee hive chemicals at label rates in field conditions.

The number of queen cells under construction was significantly affected by hive chemical regime, whilst it was not significantly affected by the mite covariate. The number of queen cells under construction was significantly higher in colonies receiving bee hive chemicals than in non-treated controls. We included this variable as a proxy measure of the queen’s state, as her supersedure is generally considered an indicator of suboptimal distribution of queen mandibular pheromone within the colony [31]. Without suggesting a mechanism, our results indicate that exotic chemicals in the nest matrix are associated with higher rates of queen replacement.

Adult bee population (frames of bees) was not significantly affected by hive chemical regime after adjusting for the mite covariate. However, the effect of the covariate was significantly negative so that increasing mites were associated with decreasing bee populations, an effect shown before [32]. There was a significant interaction between chemical regime and the mite covariate; however, the direction of the effect was always negative, whether for the three chemicals (*P*<0.01 in each case) or untreated control (*P* = 0.025). Therefore, mite levels were influential in these results, but they varied independently of colony chemical treatment (see section 3.1). Our most important finding here is that bee hive chemicals, in isolation from confounding effects of mites, did not affect colony bee populations.

The amount of brood (frames of brood) was not significantly affected by hive chemical regime after adjusting for the mite covariate. However, the effect of the covariate was significantly negative so that the amount of brood decreased as mite level increased, a phenomenon known from previous authors [32]. There was a significant interaction between chemical regime and the mite covariate; however, the direction of the effect was always negative, whether for the three chemicals (*P*<0.01 in each case) or untreated control (*P* = 0.049).

Time (sec) for marked, released bees to return to the nest was not significantly affected by hive chemical regime after adjusting for the mite covariate. However, the effect of the covariate was significant and negative so that the length of time for a bee to return to the nest decreased as colony mite level increased. These results stand in contrast to earlier experiments dedicated to the hypothesis that phoretic mites affect homing behavior of foraging bees [33]. In that work, foragers were released at different distances, and mite-infested individuals took over twice as long as non-infested individuals to return to their nests. The differences in our designs are considerable and include different release distances (5–400 m *vs*. 500 m in present study) and comparisons of individual mite levels [33] vs. colony mite levels (present study). These are enough to render comparisons difficult, but the collective evidence suggests that Varroa may act differently on individual behaviors vs. mean colony effects. For our present purposes, however, we have no evidence that bee hive chemicals at field rates affected honey bee homing.

The incidence of colony Nosema spore loads scoring “medium,” “medium high,” or “high” was not significantly affected by hive chemical regime after adjusting for the mite covariate. However, the effect of the covariate was significantly positive so that spore count increased as colony mite level increased. A similar correlative association was shown in Argentina where investigators found that colonies more heavily loaded with Varroa sustained higher Nosema spore loads after the seasonal peak in spore formation occurred [34]. This contributes to a mounting database that managed honey bees are increasingly subject to multiple stressors [35]. But our main conclusion here is that bee hive chemicals at field rates did not significantly affect colony Nosema spore load.

### Conditioned Learning and Memory

Results are shown in Table 3. No mite covariate was included in these analyses because we were forced to pool colonies by treatment, state, year, and season to create one replicate due to small numbers of responding bees in some colonies; therefore, we could not associate response data to unique colony mite levels. There were no significant effects of chemical hive treatment nor post-conditioning time interval (7, 14, 28, or 56 min) on the percentage of bees expressing retained memory from the learning conditioning trials (*P* = 0.39). For percentage of bees learning, however, there were significant (*P*<0.001) effects of hive chemical regime on learning trials 2–5 (trial 1 was discarded as described in Methods). But despite the significant effect of treatment as shown by the chi-square value, it is not possible to identify which groups are significantly different from one another. Nevertheless, bees from the Apistan-treated group performed comparatively well. These results provide field-level context to earlier work on the effects of acaricides on honey bee cognition. Although topical applications of fluvalinate at sublethal rates are known to reduce movement of individuals and their social exchanges with nest-mates [36], there is no similar evidence for an effect of fluvalinate on bee response to the PER assay [37]. This raises the possibility that the present results are indicating heightened cognitive performance by bees for whom Varroa control has been optimized by fluvalinate. For these PER data we were not able to partition out a mite covariate; however it is worth noting that mite control, although never different from non-treated controls, was nevertheless optimized in colonies receiving Apistan™ (Table 2). This interpretation is consistent with evidence that Varroa parasitism changes the expression of genes responsible for host embryonic development and that bees known to be mite tolerant have measurable differences in the expression of genes controlling neuronal development and sensitivity [38].

### Adult Bee Emergence Weight and Longevity

**Figure 1:**
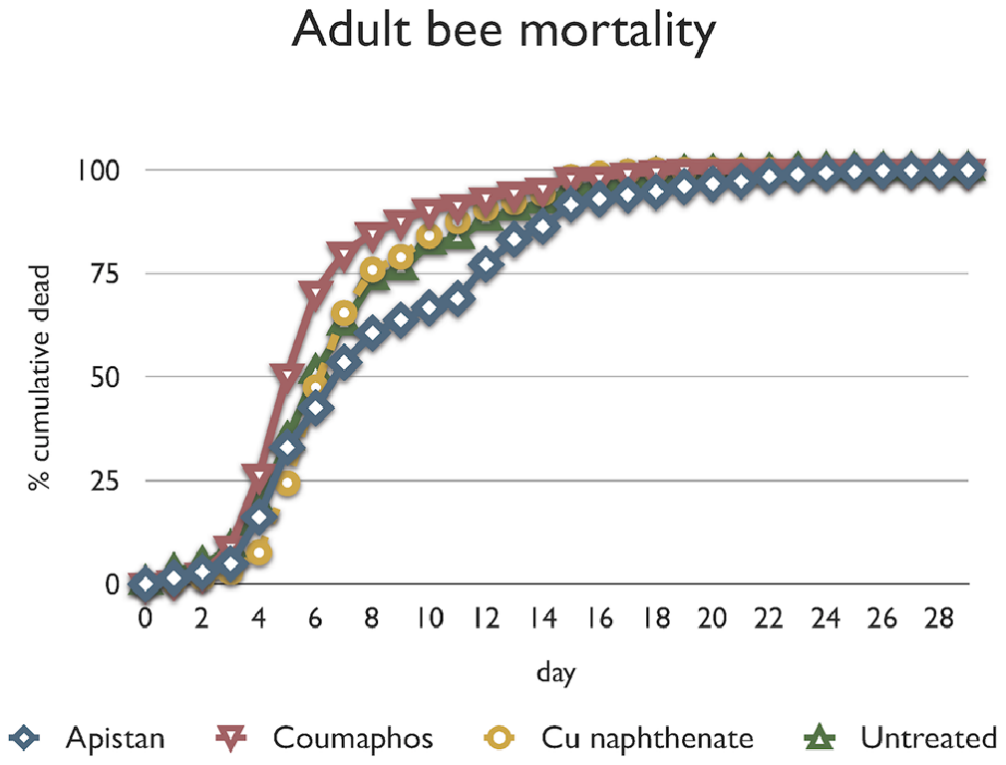
Cumulative daily adult bee mortality. Same-aged cohorts were made in the lab from adult bees reared as brood in field colonies receiving labeled rates of bee hive chemicals. The cohorts were followed for cumulative daily mortality. The figure shows plotted raw data, not the proportional hazard curves fitted in the model.

Results are shown in Table 3 and Figure 1. Adult bee emergence weight (mg) was significantly affected by hive chemical regime after adjusting for the mite covariate. The effect of the covariate was significantly negative so that increasing mite levels were associated with decreasing bee weight. Bee weight was significantly higher in colonies treated with Check Mite+ or copper naphthenate than in non-treated controls; colonies treated with Apistan were intermediate. Decreased bee weight has long been known to be an artifact of Varroa parasisitm [39], and these data are weak evidence for a measure of mite mitigation with Check Mite+ (but see section 3.1).

Cumulative daily mortality was analyzed by analysis of deviance using a hazard function – the probability of an individual dying at a fixed time point relative to a control group baseline. Hazard function was significantly affected by hive chemical regimen (change of deviance = 202.5; df = 3; *P*<0.001), whilst the mite covariate did not have any significant effect on the hazard function (change of deviance = 0.29; df = 1; *P* = 0.59). Pairwise separation tests of the three test chemicals (Apistan, Check Mite+, and Cu napthenate) showed that hazard function followed the pattern Check Mite+>(Cu naphthenate ≅ non-treated control)>Apistan. This pattern of comparative mortality is graphically apparent in Figure 1 where near the middle of the curve the cumulative daily mortality spread among the three chemicals is most divergent and the control group is mid-point. These data, adjusted for the mite covariate, are unambiguous evidence that bee hive chemicals are associated with legacy survival effects on the bees exposed to them as immatures. Check Mite+ caused higher legacy mortality than non-treated controls, and Apistan improved legacy mortality relative to non-treated controls; however, evidence that these two molecules synergize to cause lethal effects in bees [8]strengthens the argument for honey bee health management approaches that deemphasize synthetic miticides.

## Summary and Conclusions

Exotic chemicals are routinely and legally inserted into hive matrices as part of honey bee health management strategies. Key to understanding the effects of these chemicals on the host is disambiguating their sublethal effects from their purported benefits – in our case, killing parasitic Varroa mites. We attempted to do this by adjusting our analyses for a continuous covariate – a colony Varroa level index. We included copper naphthenate wood preservative as an outgroup chemical. Even though it has no known or suspected miticidal properties, we nevertheless subjected it to covariate adjustment so we could unambiguously compare it alongside the miticides for its impact on bees.

After adjusting for the mite covariate, exotic hive chemicals significantly decreased 3-day brood survivorship and increased construction of queen supercedure cells compared to non-treated controls. Bees exposed to Check Mite+ as immatures had higher legacy mortality as adults relative to non-treated controls, whereas bees exposed to Apistan had improved legacy mortality relative to non-treated controls; bees exposed to Cu naphthenate were intermediate and not significantly different from controls. In contrast to these morbidities, Check Mite+ significantly improved adult emergence weight over non-treated controls, and Apistan-treated bees performed comparatively well on tests of associative learning. And finally, there were no effects of bee hive chemical detected for frames of bees, frames of brood, frames of honey, foraging rate, time required for marked released bees to return to their nest, percentage of released bees that return to the nest, and colony Nosema spore loads.

To our knowledge, this is the first study to examine sublethal effects of bee hive chemicals applied at label rates under field conditions while disambiguating the results from any mite control benefits realized from the chemicals. Given the poor performance of the miticides at reducing mite levels and their inconsistent effects on the host, these results emphasize the importance of minimizing use of exotic hive chemicals in honey bee management.

## References

[1] Rinderer TE, DeGuzman L, Lancaster V, Delatte G, Stelzer JA (1999) Varroa in the mating yard: The effects of *Varroa jacobsoni* and Apistan on drone honey bees. Am Bee J 139: 134–139.

[2] Burley LM, Fell RD, Saacke RG (2008) Survival of honey bee (Hymenoptera: Apidae) spermatozoa incubated at room temperature from drones exposed to miticides. J Econ Entomol 101: 1081–1087. 18767713 10.1603/0022-0493(2008)101[1081:sohbha]2.0.co;2

[3] Haarmann T, Spivak M, Weaver D, Weaver B, Glenn T (2002) Effects of fluvalinate and coumaphos on queen honey bees (Hymenoptera: Apidae) in two commercial queen rearing operations. J Econ Entomol 95: 28–35 DOI 10.1603/0022-0493-95.1.28. 11942761

[4] Pettis JS, Collins AM, Wilbanks R, Feldlaufer MF (2004) Effects of coumaphos on queen rearing in the honey bee, *Apis mellifera* . Apidologie 35: 605–610.

[5] Collins AM, Pettis JS, Wilbanks R, Feldlaufer MF (2004) Performance of honey bee (*Apis mellifera*) queens reared in beeswax cells impregnated with coumaphos. J Apic Res 43: 128–134.

[6] Boncristiani H, Underwood R, Schwarz R, Evans JD, Pettis J, et al (2011) Direct effect of acaricides on pathogen loads and gene expression levels in honey bees *Apis mellifera* . J Insect Physiol 58: 613–620. 22212860 10.1016/j.jinsphys.2011.12.011

[7] Locke B, Forsgren E, Fries I, de Miranda J (2012) Acaricide treatment affects viral dynamics in *Varroa destructor*-infested honey bee colonies via both host physiology and mite control. Appl Environ Microbiol 78(1): 227–235 10.1128/AEM.06094-11 22020517 PMC3255632

[8] Johnson RM, Pollock HS, Berenbaum MR (2009) Synergistic interactions between in-hive miticides in *Apis mellifera* . J Econ Entomol 102: 474–479. 19449624 10.1603/029.102.0202

[9] Johnson RM, Dahlgren L, Siegfried BD, Ellis MD (2013) Acaricide, fungicide and drug interactions in honey bees (*Apis mellifera*). PLoS ONE 8(1): e54092 10.1371/journal.pone.0054092 23382869 PMC3558502

[10] vanEngelsdorp D, Hayes J Jr, Underwood RM, Pettis JS (2010) A survey of honey bee colony losses in the United States, fall 2008 to spring 2009. J Apic Res 49: 7–14.

[11] Potts SG, Roberts SPM, Dean R, Marris G, Brown MA, et al (2010) Declines of managed honey bees and beekeepers in Europe. J Apic Res 49: 15–22.

[12] Chauzat M-P, Faucon J-P, Martel A-C, Lachaize J, Cougoule N, et al (2006) A survey of pesticide residues in pollen loads collected by honey bees in France. J Econ Entomol 99: 253–262. 16686121 10.1603/0022-0493-99.2.253

[13] Mullin CA, Frazier M, Frazier JL, Ashcraft S, Simonds R, et al. (2010) High levels of miticides and agrochemicals in North American apiaries: Implications for honey bee health. PLoS ONE 5, e9754. 10.1371/journal.pone.0009754 PMC2841636 20333298

[14] vanEngelsdorp D, Evans JD, Saegerman C, Mullin C, Haubruge E, et al. (2009) Colony collapse disorder: A descriptive study. PLoS ONE 4, e6481. 10.1371/journal.pone.0006481 PMC2715894 19649264

[15] vanEngelsdorp D, Speybroeck N, Evans JD, Nguyen BK, Mullin C, et al (2010) Weighing risk factors associated with bee colony collapse disorder by classificiation and regression tree analysis. J Econ Entomol 103: 1517–1523. 21061948 10.1603/ec09429

[16] Genersch E, von der Ohe W, Kaatz H, Schroeder A, Otten C, et al (2010) The German bee monitoring project: a long term study to understand periodically high winter losses of honey bee colonies. Apidologie 41: 332–352.

[17] Burgett M, Burikam I (1985) Number of adult honey bees (Hymenoptera: Apidae) occupying a comb: a standard for estimating colony populations. J Econ Entomol 78: 1154–1156.

[18] Skinner JJ, Parkman JP, Studer MD (2001) Evaluation of honey bee miticides, including temporal and thermal effects on formic acid gel vapours, in the central south-eastern USA. J Apic Res 40: 81–89.

[19] Shimanuki H, Knox DA (2000) Diagnosis of honey bee diseases. US Dept Agric. Handbook AH-690: 61.

[20] Doull KM (1965) The effects of time of day and method of sampling on the determination of *Nosema* disease in beehives. J Invertebr Pathol 7: 1–4.

[21] Abramson CI, Sokolowski MBC, Wells H (2011) Issues in the study of proboscis conditioning. In: Stewart, EM, editor. Social insects: Structure, function, and behavior. Nova Science Publishers. 25–49.

[22] Picard-Nizou AL, Grison R, Olsen L, Pioche C, Arnold G, et al (1997) Impact of proteins used in plant genetic engineering: toxicity and behavioral study in the honeybee. J Econ Entomol 90(6): 1710–1716.

[23] Smith BH (1991) The olfactory memory of the honeybee *Apis mellifera*. I. Odorant modulation of short- and intermediate-term memory after single-trial conditioning. J Exp Biol 161: 367–382.

[24] VSN International (2012) GenStat for Windows 15th Edition. VSN International, Hemel Hempstead, UK. Available: http://www.GenStat.co.uk.

[25] McCullagh P, Nelder J (1989) Generalized Linear Models, Second Edition. Boca Raton: Chapman and Hall.

[26] Hardin J, Hilbe J (2003) Generalized Estimating Equations. London: Chapman and Hall.

[27] Cox DR (1972) Regression models and life-tables. Journal of the Royal Statistical Society. Series B (Methodological) 34(2): 187–220.

[28] Elzen PJ, Baxter JR, Spivak M, Wilson WT (2000) Control of *Varroa jacobsoni* Oud. resistant to fluvalinate and amitraz using coumaphos. Apidologie 31: 437–441 DOI 10.1051/apido:2000134.

[29] Pettis JS (2004) A scientific note on *Varroa destructor* resistance to coumaphos in the United States. Apidologie 35: 91–92 DOI 10.1051/apido:2003060.

[30] Wu JY, Anelli CM, Sheppard WS (2011) Sub-lethal effects of pesticide residues in brood comb on worker honey bee (*Apis mellifera*) development and longevity. PLoS ONE 6(2): e14720 10.1371/journal.pone.0014720 21373182 PMC3044129

[31] Butler CG (1957) The process of queen supersedure in colonies of honeybees (*Apis mellifera* Linn.). Insectes Sociaux 4: 211–223.

[32] Murilhas AM (2002) *Varroa destructor* infestation impact on *Apis mellifera carnica* capped worker brood production, bee population and honey storage in a Mediterranean climate. Apidologie 33: 271–281 10.1051/apido:2002013

[33] Kralj J, Fuchs S (2006) Parasitic *Varroa destructor* mites influence flight duration and homing ability of infested *Apis mellifera* foragers. Apidologie 37: 577–587 10.1051/apido:2006040

[34] Mariana F, Maggi M, Porrini M, Fuselli S, Caraballo G, et al (2012) Parasitic interactions between *Nosema* spp. and *Varroa destructor* in *Apis mellifera* colonies. Zootecnia Trop 30: 81–90.

[35] Cornman RS, Tarpy DR, Chen Y, Jeffreys L, Lopez D, et al (2012) Pathogen webs in collapsing honey bee colonies. PLoS ONE 7(8): e43562 10.1371/journal.pone.0043562 22927991 PMC3424165

[36] Teeters BS, Johnson RM, Ellis MD, Siegfried BD (2012) Using video-tracking to assess sublethal effects of pesticides on honey bees (*Apis mellifera* L.). Environ Toxicol Chem 31: 1349–1354 10.1002/etc.1830 22488825

[37] Decourtye A, Devillers J, Genecque E, Le Menach K, Budzinski H, et al (2005) Comparative sublethal toxicity of nine pesticides on olfactory learning performances of the honeybee *Apis mellifera* . Arch Environ Contam Toxicol 48: 242–250. 15750780 10.1007/s00244-003-0262-7

[38] Navajas M, Migeon A, Alaux C, Martin-Magniette ML, Robinson GE, et al (2008) Differential gene expression of the honey bee *Apis mellifera* associated with *Varroa destructor* infection. BMC Genomics 9: 301 10.1186/1471-2164-9-301 18578863 PMC2447852

[39] De Jong D, De Jong PH, Gonçalves LS (1982) Weight loss and other damage to developing worker honey bees from infestation with *Varroa jacobsoni* . J Apic Res 21: 165–167.

